# Association between Urine Phthalate Levels and Poor Attentional Performance in Children with Attention-Deficit Hyperactivity Disorder with Evidence of Dopamine Gene-Phthalate Interaction

**DOI:** 10.3390/ijerph110706743

**Published:** 2014-06-27

**Authors:** Subin Park, Bung-Nyun Kim, Soo-Churl Cho, Yeni Kim, Jae-Won Kim, Ju-Young Lee, Soon-Beom Hong, Min-Sup Shin, Hee Jeong Yoo, Hosub Im, Jae Hoon Cheong, Doug Hyun Han

**Affiliations:** 1 Department of Psychiatry, Seoul National Hospital, Seoul 110-744, Korea; E-Mails: subin-21@hanmail.net (S.P.); yeni1004@gmail.com (Y.K.); 2 College of Medicine and Behavioral Medicine Institute, Seoul National University, Seoul 110-744, Korea; E-Mails: soochurl@snu.ac.kr (S.-C.C.); adore412@paran.com (J.-W.K.); sabina93@hanmail.net (J.-Y.L.); hsbmdmore@hanmail.net (S.-B.H.); Shinms@snu.ac.kr (M.-S.S.); hjyoo@snu.ac.kr (H.J.Y.); 3 Center for Life and Environmental Science, Neodin Medical Institute, Seoul 110-744, Korea; E-Mail: hygiene@neodin.com; 4 Uimyung Research Institute for Neuroscience, Sahmyook University, Seoul 110-744, Korea; E-Mail: cheongjh@syu.ac.kr; 5 Department of Psychiatry, College of Medicine, Chung Ang University, Seoul 110-744, Korea; E-Mail: hduk@dreamwiz.com

**Keywords:** phthalate, attention-deficit hyperactivity disorder, neuropsychology, dopamine

## Abstract

Although there is some evidence supporting the existence of an association between prenatal maternal or postnatal child’s urine phthalate metabolite concentrations and poor attentional performances, the interaction between urine phthalate metabolite levels and genetic variation for neuropsychological deficit of attention-deficit hyperactivity disorder (ADHD) has not been examined. The aim of this study was to determine whether phthalate metabolites in urine are associated with poor neuropsychological performance in children with ADHD, and whether such association is affected by genotype-phthalate interaction. A cross-sectional examination of urine phthalate metabolite concentrations and the continuous performance test (CPT) were performed in 179 Korean children with ADHD recruited from department of psychiatry of university hospital. Correlations between urine phthalate metabolite concentrations and the CPT scores were investigated, and the interaction of phthalate metabolite levels with the selected polymorphisms at major candidate genes for ADHD, namely dopamine receptor D4 (DRD4), dopamine transporter, α-2A-adrenergic receptor, and norepinephrine transporter genes. For the subjects with the DRD4 4/4 genotype, there were significant associations of the urine phthalate metabolite concentrations with the number of omission errors, the number of commission errors, and the response time variability scores on the CPT. However, for the subjects without the DRD4 4/4 genotype, no significant associations were found. The results of this study suggest a possible association between phthalate metabolite concentrations and poor attentional performances of ADHD as well as a genetic influence on this association. Further prospective and epigenetic studies are needed to investigate causality and pathophysiological mechanisms.

## 1. Introduction

Phthalates are a group of synthetic plasticizers and solvents that are used in a wide variety of commercial products, including food packaging, polyvinyl chloride tubing, medical equipment, toys, and cosmetics. Due to their high-production volume, common use, and widespread environmental contamination, these compounds reach humans through ingestion, inhalation, and dermal exposure daily [1]. Phthalate esters are manufactured from alcohols ranging from methanol and ethanol (C1/C2) up to *iso*-tridecanol (C13), either as a straight chain or with some branching. They are divided into two distinct groups, with different applications, toxicological properties, and classification, based on the number of carbon atoms in their alcohol backbone. High molecular weight (HMW) phthalates, or high phthalates, include those with 7–13 carbons in their backbone, which gives them more permanency and durability. Low molecular weight phthalates (LMW), or low phthalates, are those with only 3–7 carbon molecules in their backbones [2]. European Union risk assessments showed positive results regarding the safe use of high phthalates (diisononyl phthalate (DINP), diisodecyl phthalate (DIDP), di(2-propylheptyl) phthalate (DPHP), diisoundecyl phthalate (DIUP), and ditridecyl phthalate (DTDP)). They have all been registered under Registration, Evaluation, Authorization and Restriction of Chemicals (REACH) and do not require any classification for health and environmental effects, nor are they on the candidate list for authorization [2]. In the while, LMW phthalates (di(2-ethylhexyl) phthalate (DEHP), di-*n*-butyl phthalate (DBP), diisobutyl phthalate (DIBP), and benzylbutyl phthalate (BBP)) are classified as category 1B reproductive agents (Regulation (EC) No. 1272/2008) [3] and these four phthalates are now on the REACH candidate list of substances of very high concern for authorization [4]. Phthalates exhibit distinguished differences in toxicity according to their metabolites. Especially, mono(2-ethylhexyl) phthalate (MEHP), the active monoester metabolite of DEHP, has been regarded as responsible for much of DEHP’s toxicity and may be more potent than its parent compound in toxicity [5,6].

LMW phthalates are endocrine disruptors that show estrogen-like activity. There is a growing body of evidence showing that exposure to environmental endocrine disruptors such as phthalates may adversely impact child development (e.g., fetal growth, early reproductive tract development, pubertal development, and neurodevelopment) [7]. Experimental studies suggest that higher levels of DEHP may have adverse effects on neurobehavioral parameters in mice [8,9]. In humans, prenatal exposure to phthalates has been associated with poor birth outcomes [10], adverse neurological outcomes in infants [11,12], reduced masculine play in boys [13], and social impairment [14] in childhood.

In particular, recent evidence supports the existence of an association between urine phthalate metabolite levels, attention-deficit/hyperactivity disorder (ADHD), and executive functioning. ADHD is a disorder primarily characterized by inattention, impulsivity, and hyperactivity, with a worldwide prevalence of 5.3% [15]. Previous animal studies have reported that the phthalate compound might cause hyperactivity and impulsivity in rats [16]. Those animal behaviors are strikingly similar to the clinical syndrome of ADHD found in children. In the recent epidemiological studies, it has been reported that prenatal maternal [17] and postnatal child’s [18,19] urine phthalate metabolite levels were associated with ADHD symptoms and poor executive functioning. However, no studies have been conducted in children who have been confirmed diagnosis of ADHD through meticulous diagnostic process.

ADHD is a multi-factorial polygenic disorder, and evidence points to dysregulation of the dopaminergic and noradrenergic system being involved in the pathophysiology of ADHD [20]. Its leading candidate genes include those involved in dopaminergic or noradrenergic neurotransmission [20]. The variable number of tandem repeat (VNTR) located on the 3′-untranslated region (3′-UTR) of the dopamine transporter gene (DAT1) and the VNTR in exon III of the dopamine D4 receptor gene (DRD4) are the two polymorphic systems most studied and most frequently replicated in relation to ADHD.

The genetic heritability of ADHD is reported to be approximately 75%, and environmental factors are estimated to account for 25% of the development of ADHD [21]. Recent studies suggest that genetic influences might function to increase the susceptibility of the brain to environmental stressors such as environmental hormones [22], and such gene-environmental interaction might affect deficits in executive functioning and attentional processes, which are supposed to be the central pathophysiology of ADHD. Previous animal study using DNA microarray data have found that phthalate metabolites change the expression patterns of the DRD4 and DAT1 in the midbrain [16].

In this study, to elucidate the contribution of phthalates to the pathophysiological mechanisms leading to ADHD, we investigated urine phthalate metabolite levels with regard to their relationships with attentional performances in a sample of children with ADHD. The interaction of urine phthalate metabolite levels with the selected polymorphisms at two major candidate genes for ADHD, namely DRD4 andDAT1 was also examined in relation to neuropsychological measures.

## 2. Methods

### 2.1. Participants

We recruited 179 children with ADHD from department of psychiatry of university hospital in South Korea. The recruited children were between 6 and 15 years old and had been diagnosed with ADHD according to Diagnostic and Statistical Manual of Mental Disorders, 4th Revision (DSM-IV) criteria as ascertained by a child psychiatrist. Exclusion criteria included the following: (1) a history of pervasive developmental disorder, mental retardation, bipolar disorder, psychotic disorder, obsessive compulsive disorder, or Tourette’s syndrome; (2) a history of organic brain disease, seizure disorder, or other neurological disorder; (3) an intelligence quotient (IQ) below 70; (4) the presence of learning disabilities or language disorders; (5) the presence of major depressive disorder, anxiety disorder, or tic disorder requiring drug therapy; and (6) any previous course of methylphenidate treatment lasting more than 1 year or occurring within the last 4 weeks. Children with ADHD, prescribed methylphenidate, were excluded due to potential confounding neuropsychological effects. To diagnose ADHD and any comorbid disorders, we used the Korean Kiddie-Schedule for Affective Disorders and Schizophrenia—Present and Lifetime Version (K-SADS-PL) [23]. The Korean version of K-SADS-PL was reported to be valid and reliable for diagnosing major child psychiatric disorders in Korean children (consensual validity, kappa value = 0.695; test-retest reliability, kappa value = 0.755; sensitivity = 0.774; specificity = 0.947 for ADHD) [23]. We assessed intellectual abilities using the abbreviated form of the Korean Educational Development Institute’s Wechsler Intelligence Scales for Children [24]. We provided detailed information about the study to the parents and children and then obtained written informed consent before any child entered the study. The study protocol was approved by the institutional review board of the Seoul National University Hospital.

### 2.2. Assessment of the Neuropsychological Functions

The children with ADHD were administered the Continuous Performance Test (CPT). The CPT is the neuropsychological test most widely used to measure inattention and impulsivity among children with ADHD. The Korean version of the CPT was standardized, and its validity and reliability are well established [25]. Three variables were recorded: (1) the number of omission errors (failures to respond to the target), which are commonly interpreted as indicators of inattention; (2) the number of commission errors (inappropriate responses to the non-target), which are commonly interpreted as indicators of impulsivity; and (3) the standard deviations of the response times for correct responses to the target (response time variability), which are interpreted as indicators of variability or consistency of attention. The raw data of three test variables are converted to aged-adjusted T-scores, which are scaled to have a mean of 50 and a standard deviation of 10. Higher T-scores indicated worse test performance.

### 2.3. Phthalate MetaboliteLevel Measurements

The spot urine samples were collected in 50 mL sterile specimen containers at the outpatient clinics in the morning and were refrigerated at −20 °C immediately. Refrigerated specimens were transported to the laboratory within 2 h. We measured the metabolites of DEHP and DBP among several phthalate compounds, because they were most widely used phthalates in personal-care products (e.g., DEHP in various kinds of plastic products, including vinyl flooring, paints, toys and plastic bags and DBP in cosmetic products such as perfume, aftershaves, and shampoo) and have been most widely linked to adverse health effects [5]. DEHP is metabolized to the primary metabolite MEHP and subsequently to secondary metabolites, mono(2-ethyl-5-hydroxyhexyl) phthalate (MEHHP) mono(2-ethyl-5-oxohexyl) phthalate (MEOP), and mono-(2-ethyl-5-hydroxylhexyl) phthalate (MEHHP), and DBP is metabolized to the primary metabolite mono-n-butyl phthalate (MBP) [5]. The metabolites measured in this study included MEHP, MEOP, and MBP, because these metabolites have been reported to be associated with cognitive function and behaviors of children in previous studies [18,19]. The urine phthalate metabolite concentrations were measured with high-performance liquid chromatography tandem mass spectrometry (Agilent 6410 triple Quad LCMS; Agilent Technologies, Santa Clara, CA, USA). Five hundred microliters of urine were buffered with 30 µL of 2.0 mol/L sodium acetate (pH 5.0) and then spiked with a mixture of isotope phthalate monoester standards (100 ng/mL) and 10 µL of β-glucuronidase. The sample was incubated at 37 °C for 3 h to deconjugate the glucuronidated phthalate metabolites. After incubation, 100 µL of 2 nmol/L hydrogen chloride was added to collect phthalate monoester. The extract was dried with nitrogen gas and reconstituted with 1 mL of high-performance liquid chromatography-grade water in a 2-mL glass vial. One blank and one quality control (QC) sample were included in each batch of samples. The QC sample was spiked with pooled urine and a mixture of phthalate monoester standard (100 ng/mL). The supernatants were purified by solid phase extraction with disposable AgilentC18 1.8 µm (2.1 × 50 mm). The mobile phase was 0.1% acetic acid water: 0.01% acetic acid acetonitrile (90:10, *v/v*) at a flow rate of 0.25 mL/min, and the eluates were monitored at target masses of 221, 293, and 291 and internal standard masses of 225, 297, and 295 [18]. We used the value (micrograms per liter)/creatinine (grams per liter) for dilution correction in the analyses. Because the concentrations were not normally distributed, we used their natural log-transformed values.

### 2.4. Genotyping

Genomic DNA was extracted from blood lymphocytes using a Genomic DNA Extraction Kit (Bioneer, Korea). DAT1 was localized on chromosome 5p15.3. The 40-base pair VNTR polymorphism located in the 3′-UTR of DAT1 was genotyped, as previously described [26]. T7-5 Long (5′-TGT GGT GTA GGG AAC GGC CTG AG-3′) and T7-3a Long (5′-CTT CCT GGA GGT CAC GGC TCA AGG-3′) were used at concentrations of 5 × 10 − 7 M. For the DRD4 exon III VNTR polymorphism [27], the oligonucleotide primers (5′-ACC ACC ACC GGC AGG ACC CTC ATG GCC TTG CGC TC-3′ and 5′-CTT CCT ACC CTG CCC GCT CAT GCT GCTGCT CTA CTG G-3′) were used to generate the DRD4 exon III polymorphic region (2–10 variable repeat units, 1 unit = 48 base pairs).

### 2.5. Statistical Analysis

Correlations between the MEHP, MEOP, and MBP concentrations were examined using Pearson correlation analyses. We conducted linear regression analyses to elucidate the associations between the urine phthalate metabolite concentrations and the neuropsychological variables. Nine set of regression analyses were performed using the (ln) concentrations of MEHP, MEOP, or MBP (continuous, μg/g) as a principal predictor and each of the individual CPT measures (*i.e.*, omission errors, commission errors, or response time variability) (continuous, T-scores) as an outcome. All regression models included a set of covariates based on established predictors of children’s cognitive and behavioral functioning: age (continuous, in years), gender (male *vs.* female), years of parental education (continuous, in years), yearly family income (above $25,000 *vs.* below $25,000), subtype of ADHD (inattentive, hyperactive-impulsive, combined, not otherwise specified), and IQ (continuous). The categorical variable (*i.e.*, gender, yearly family income, and subtype of ADHD) was incorporated in the linear regression analyses using dummy variable coding. All variables were concurrently entered into the model. Next, we conducted multivariate modeling to examine the relationships among the urine phthalate metabolite concentrations, DRD4 or DAT1 genotypes, and neuropsychological variables while including age, gender, years of parents’ education, yearly income, subtype of ADHD, and IQ as covariates. A genotype-by-phthalate metabolite concentration interaction term was also included in the model. In these models, the genotype variable dichotomized subjects into two groups. Thus, the subjects were dichotomized into those with the DRD4 4/4 or the DAT1 10/10 genotype and those with another genotype. This differentiation was selected because previous studies have reported an association between the 4-repeat allele homozygote of DRD4 with clinical and neuropsychological phenotypes of ADHD [27,28] and an association between the 10-repeat allele homozygote of DAT1 with ADHD susceptibility and poor treatment response to methylphenidate in Korean populations [26,29]. Subsequent multiple regression analyses were performed using the urine phthalate metabolite concentrations as a principal predictor and the neuropsychological variables as an outcome in ADHD children with the DRD4 4/4 genotype and in those with another genotype, separately. All statistical analyses were performed using SPSS (version 21.0; SPSS Inc., Chicago, IL, USA), with statistical significance defined as alpha level <0.025 (=0.05/2SNPs).

## 3. Results

### 3.1. Characteristics of Participants

The 179 clinic-referred children with ADHD (147 males and 32 girls, ages 6–15 years, mean age 8.98 ± 2.39 years) participated in the study between September 2010 and June 2012. Table 1 shows the demographic and clinical characteristics of the participants. The combined type was the most common in our sample (46.4%), followed by the inattentive (38.0%) type. The most common comorbidity was oppositional defiant disorder (12.2%).

**Table 1:** Characteristics and genetic polymorphism of the DRD4 gene of the children with ADHD (*n* = 179).

Variable	ADHD (n = 179)
Gender, male/female, No. (%)	147/32 (82.1/17.9)
Age, mean (SD), years	8.98 (2.39)
ADHD subtype, No. (%)	
Combined	83 (46.4)
Predominantly inattentive	68 (38.0)
Predominantly hyperactive-impulsive	7 (3.9)
Not otherwise specified	21 (11.7)
Psychiatric comorbidity, No. (%)	
Oppositional defiant disorder	22 (12.2)
Anxiety disorder	4 (2.2)
Tic disorder	5 (2.8)
Enuresis	4 (2.2)
Intelligence Quotient, mean (SD)	106.21 (14.43)

Note: ADHD, attention deficit hyperactivity disorder.

The distributions of the genotypes were all in agreement with the expected values of the Hardy characteristic equilibrium (*p >* 0.05). Among the genotypes of DRD4, the 4/4 genotype was observed in 96 (53.6%) out of 179 samples, the 2/4 genotype in 56 (31.3%), the 4/5 genotype in 14 (7.8%), and the 2/2 genotype in five (2.8%) each. The 2/5 and 3/4 genotypes were observed in three (1.7%) each and the 5/5 and 5/7 genotypes in one (0.6%). Among the genotypes of DAT1, the 10/10 genotype was observed in 148 (82.7%) out of 179 samples and the 9/10 genotype and the 7/10 genotype were observed in 10 (5.6%) each. The 10/11 genotype was observed in seven (3.9%), the 7/7 genotype in two (1.1%), and the 6/9 and the 6/10 genotype in one (0.6%).

All of the urinary phthalate biomarkers exceeded the limit of detection. The mean geometric mean (ln) concentrations of creatinine-corrected MEHP, MEOP, and MBP were 45.60 μg/g (geometric SD (GSD) = 2.41; range: 2.95–892.65), 43.82 μg/g (GSD = 2.18; range: 3.70–785.44), and 68.03 μg/g (GSD = 2.10; range, 91.90–109.76), respectively. Urine MEHP, MEOP, and MBP concentrations were highly correlated with each other (Table 2).

**Table 2:** Associations between the urine phthalate metabolite concentrations and the continuous performance test variables.

CPT	MEHP		MEOP		MBP	
	*B* (95% CI)	*p*	*B* (95% CI)	*p*	*B* (95% CI)	*p*
Omission errors	8.46 (−0.96–17.89)	0.078	8.05 (−2.61–18.70)	0.137	6.28 (−5.34–17.89)	0.287
Commission errors	9.97 (0.81–19.14)	0.033	15.52 (5.36–25.69)	0.003	3.44 (−7.95–14.83)	0.552
Response time variability	6.88 (0.80–12.95)	0.027	7.67 (0.82–14.52)	0.028	5.97 (−1.53–13.47)	0.118

Notes: Adjusted for age, gender, years of paternal and maternal education, yearly income, subtype, and intelligence quotient; Abbreviations: CPT, continuous performance test; MEHP, mono-2-ethylhexyl phthalate; MEOP, mono(2-ethyl-5-oxohexyl)phthalate; MBP, mono-n-butyl phthalate; *B*, unstandardized regression coefficient; CI, confidence intervals.

### 3.2. Associations between the Phthalate Metabolite Concentrations and the CPT Variable Scores

After adjusting for age, gender, years of parents’ education, yearly income, subtype of ADHD, and IQ, the number of commission errors was positively and significantly associated with the MEOP concentrations (unstandardized regression coefficient, *B* = 15.52, 95% CI = 5.36–25.69 for a 1-unit increase in natural log-transformed urine MEOP concentration) and response time scores were positively and significantly associated with the MBP concentrations (*B =* 8.16, 95% CI = 1.52–14.80 for a 1-unit increase in natural log-transformed urine MBP concentration) (Table 3).

### 3.3. Effects of the Phthalate Metabolite Concentration by DRD4 Genotype on the CPT Variable Scores

After adjusting for age, gender, years of parents’ education, yearly income, subtype of ADHD, and IQ, we found a significant interaction of the MEHP concentrations with the DRD4 genotype in relation to omission errors (F = 6.57, *p =* 0.012) and commission errors (F = 5.80, *p =* 0.018). We also found a trend of an interaction of the MEOP concentrations with the DRD4 genotype in relation to omission errors (F = 4.53, *p =* 0.035) and response time variability (F = 5.17, *p =* 0.025) and a trend of an interaction of the MBP concentrations with the DRD4 genotype in relation to omission errors (F = 3.39, *p =* 0.068) (Table 3). The interaction of the phthalate metabolite concentrations with the DAT1 genotype was not statistically significant in any CPT variable models (data available on request).

**Table 3:** .ANCOVA models examining the main and interaction effects of the urine phthalate metabolite concentrations and the DRD4 genotype on the continuous performance test variables.

Model	OE		CE		RTSD	
	F	*p*	F	*p*	F	*p*
ANCOVA model 1						
DRD4 (4/4)	1.81	0.180	1.06	0.304	2.87	0.093
MEHP	0.15	0.701	0.02	0.902	0.01	0.942
MEHP by DRD4 interaction	6.57	**0.012**	5.80	**0.018**	3.24	0.074
ANCOVA model 2						
DRD4 (4/4)	0.80	0.374	0.15	0.703	3.91	0.050
MEOP	0.04	0.835	0.65	0.421	0.002	0.964
MEOP by DRD4 interaction	4.54	0.035	3.15	0.078	5.17	0.025
ANCOVA model 3						
DRD4 (4/4)	0.60	0.441	0.003	0.956	2.01	0.159
MBP	0.25	0.620	0.12	0.731	0.16	0.686
MBP by DRD4 interaction	3.39	0.068	1.10	0.296	2.00	0.160

Notes: Full analysis of covariance (ANCOVA) models include age, gender, years of paternal and maternal education, yearly income, subtype, intelligence quotient (IQ), dopamine receptor D4 (DRD4) genotype, urine phthalate metabolites concentrations, and the urine metabolite concentrations by DRD4 genotype interaction. Models had a total of 171 df; Abbreviations: OE, omission errors; CE, commission errors; RTSD, response time variability; MEHP, mono-2-ethylhexyl phthalate; MEOP, mono(2-ethyl-5-oxohexyl)phthalate; MBP, mono-*n*-butyl phthalate; Bold type: *p <* 0.025.

Next, we conducted linear regression analyses using the CPT variables as the outcome and the urine phthalate metabolite concentrations as the predictor within each genotype; thus, separate models were created for subjects with the DRD4 4/4 and other genotypes. For the subjects with the DRD4 4/4 genotype, there were positive and significant associations of the MEHP concentrations with the number of omission errors (*B =* 19.66, 95% CI = 7.38–31.95) and the response time variability scores (*B =* 11.15, 95% CI = 2.28–20.02) and of the MEOP concentrations with the number of omission errors (*B =* 21.26, 95% CI = 7.31–35.22), the number of commission errors (*B =* 21.24, 95% CI = 6.73–35.74), and the response time variability scores (*B =* 14.77, 95% CI = 4.95–24.60).

We also found a positive and significant association between the MBP concentrations and the number of omission errors (*B =* 21.96, 95% CI = 5.32–38.61) in the subjects with the DRD4 4/4 genotype. However, for the subjects without the DRD4 4/4 genotype, no significant associations were found (Table 4).

**Table 4:** Associations between the urine phthalate metabolite concentrations and the continuous performance test variables by DRD4 genotype.

CPT	MEHP						MEOP						MBP					
	DRD4 4/4			Other			DRD4 4/4			Other			DRD4 4/4			Other		
	B (S.E.)	95% CI	*p*	B (S.E.)	95% CI	*p*	B (S.E.)	95% CI	*p*	B (S.E.)	95% CI	*p*	B (S.E.)	95% CI	*p*	B (S.E.)	95% CI	*p*
OE	19.66 (6.15)	7.38–31.95	0.002	−1.19 (8.25)	−17.71–15.33	0.885	21.26 (6.99)	7.31–35.22	0.003	−4.74 (8.90)	−22.55–13.07	0.596	21.96 (8.33)	5.32–38.61	0.010	−1.76 (10.28)	−22.32–18.81	0.865
CE	14.54 (6.60)	1.35–27.73	0.031	3.06 (6.87)	−10.69–16.81	0.658	21.24 (7.26)	6.73–35.74	0.005	6.26 (7.39)	−8.53–21.05	0.400	9.34 (9.00)	−8.62–27.31	0.303	−0.02 (8.57)	−17.17–17.13	0.998
RTSD	11.15 (4.44)	2.28–20.02	0.015	2.17 (5.07)	−7.98–12.31	0.671	14.77 (4.92)	4.95–24.60	0.004	-1.04 (5.48)	−12.01–9.93	0.850	13.50 (5.93)	1.65–25.34	0.026	2.00 (6.31)	−10.64–14.63	0.753

Notes: Adjusted for age, gender, years of paternal and maternal education, yearly income, subtype, and intelligence quotient; Abbreviations: CPT, continuous performance test; DRD4, dopamine receptor 4; MEHP, mono-2-ethylhexyl phthalate; MEOP, mono(2-ethyl-5-oxohexyl)phthalate; MBP, mono-n-butyl; OE, omission errors; CE, commission errors; RTSD, response time variability; *B*, unstandardized regression coefficient; CI, confidence intervals; Bold type: *p <* 0.025.

## 4. Discussion

To the best of our knowledge, this is the first study that has identified an association between urine phthalate metabolite levels and the poor attentional performance in ADHD children with evidence of dopamine gene-phthalate interaction. We extended the findings of prior community-based studies by using a clinical sample of ADHD and by examining the interaction of urine phthalate metabolite levels with the dopaminergic and noradrenergic genes.

All of the urinary phthalate biomarkers exceeded the limit of detection in this study. Phthalate exposure is widespread in a population. Urinary phthalate metabolite levels have been shown to be stable over several months, likely due to regular, ongoing exposure to phthalate-containing products [30]. Several Korean studies showed that urinary phthalate metabolites were detected in nearly every sample [31,32,33]. Previous study reported that the concentrations of most phthalate metabolites in urines of the Korean children were greater than those reported from USA or from Germany [32].

The geometric mean concentrations of MEHP, MEOP, and MBP in clinic-referred children with ADHD in this study (45.60, 43.82, and 68.03 μg/g, respectively) were higher than those in children and adolescents aged 6 to 19 years in the previous community study of Korea (25.79, 22.20, and 45.60 μg/g, respectively) [33] and those in 6–15-year-old children in population-based survey in the USA (30.6, 21.2, and 30.2 μg/g, respectively) [19]. These results suggest higher urine phthalate metabolite concentrations in an ADHD sample than those in a community sample, but different age and gender compositions of populations across studies may affect these results.

Consistent with previous studies [18], we found a significant correlation between the urine phthalate metabolite concentrations and the poor attentional performance. The response time variability we found to be correlated with the urine phthalate metabolite concentrations is an indicator of inconsistency of attentional performance, which has been proposed as a leading endophenotype for ADHD [34]. Our results suggest that high phthalate metabolite concentrations in the body may play an important role either in the neurotransmitter system or in neurodevelopment, possibly increasing the risk of ADHD.

It is intriguing that the associations between the urine DEHP metabolite concentrations and the CPT results were observed only in subjects with the 4/4 genotype of DRD4. We could speculate that, for genetically susceptible subjects, neural substrates associated with attentional systems may be more susceptible to DEHP. The 7-repeat allele of the exon III VNTR polymorphism of the DRD4 gene—a risk allele of ADHD in European or Caucasian subjects—is known to result in a blunted response toward dopamine [35]. Asians, including Koreans, are known to rarely exhibit the 7-repeat allele of DRD4 exon III 48-bp VNTR polymorphism [27,28], and the 4-repeat allele of the exon III VNTR polymorphism of the DRD4 gene was reported to be associated with response to methylphenidate treatment in Korean children with ADHD, suggesting that person with this allele would be more susceptible to external influences. Korean children of alcoholics have been shown to be significantly more likely to carry the 4-repeat allele and the 4/4 genotype of DRD4 than are control subjects, indicating the possibility of genetic vulnerability toward alcohol-related disorders existing alongside a higher morbidity of ADHD [36].

Although the mechanism underlying a possible association between phthalates and neurodevelopment has not been established, it is possible that the toxicity of phthalates is attributable to degeneration of dopaminergic neurons, leading to the hyperkinetics observed in rats in cases of 6-hydroxydopamine procedures [37]. Indeed, animal studies have shown that phthalate metabolites significantly reduce immunoreactivity for tyrosine hydroxylase, which is a rate-limiting enzyme with regard to the production of dopamine [38]. Using DNA microarray data, researchers have found that phthalate metabolites change the expression patterns of various genes, including the DRD4 and dopamine transporter in the midbrain [16].

This study had several limitations. First, because our data were cross-sectional and correlational, no inferences about causality are possible at this time. Therefore, further research using prospective designs is needed to investigate causality. Second, there was no control group. To determine whether the influence of DRD4 genotype-phthalate metabolite levels interaction on attentional performance is specific to ADHD patients or not, we should have obtained phthalate metabolite levels and neuropsychological and genetic data from healthy controls. Third, because we excluded the children with ADHD who have been on methylphenidate treatment, the most severely affected children may be excluded from evaluation which could affect the study results. Finally, the sample size of the present study was relatively small for genotypic analysis, thus the results should be carefully interpreted.

## 5. Conclusions

In conclusion, the results of this study suggest the possibility of an association between phthalate metabolites and the severity of attentional deficits in children with ADHD and raise the issue of a genetic influence on this association. Further epigenetic studies are required to investigate the contribution of phthalates to the epigenetic pathophysiological mechanisms leading to cognitive changes.
